# What is chronic cough in children?

**DOI:** 10.3389/fphys.2014.00322

**Published:** 2014-08-28

**Authors:** Iulia Ioan, Mathias Poussel, Laurianne Coutier, Jana Plevkova, Ivan Poliacek, Donald C. Bolser, Paul W. Davenport, Jocelyne Derelle, Jan Hanacek, Milos Tatar, François Marchal, Cyril Schweitzer, Giovanni Fontana, Silvia Varechova

**Affiliations:** ^1^Service D'explorations Fonctionnelles Pédiatriques, Hôpital D'enfantsCentre Hospitalier Universitaire de Nancy, Vandoeuvre les Nancy, France; ^2^Service Des Examens de la Fonction Respiratoire et de L'aptitude à L'exerciceCentre Hospitalier Universitaire de Nancy, Vandoeuvre les Nancy, France; ^3^EA 3450 DevAH – Laboratoire de Physiologie, Faculté de Médecine, Université LorraineVandoeuvre, France; ^4^Department of Pathophysiology, Jessenius Faculty of Medicine, Comenius UniversityMartin, Slovakia; ^5^Institute of Medical Biophysics, Jessenius Faculty of Medicine, Comenius UniversityMartin, Slovakia; ^6^Department of Physiological Sciences, University of FloridaGainesville, FL, USA; ^7^Service de Médecine Infantile et de Génétique Clinique, Hôpital D'enfantsVandœuvre-lès-Nancy, France; ^8^Department of Internal Medicine, University of FlorenceFlorence, Italy

**Keywords:** child, newborn, development, plasticity of cough reflex, urge to cough and respiratory sensations, protracted bacterial bronchitis, asthma, gastro-esophageal reflux

## Abstract

The cough reflex is modulated throughout growth and development. Cough—but not expiration reflex—appears to be absent at birth, but increases with maturation. Thus, acute cough is the most frequent respiratory symptom during the first few years of life. Later on, the pubertal development seems to play a significant role in changing of the cough threshold during childhood and adolescence resulting in sex-related differences in cough reflex sensitivity in adulthood. Asthma is the major cause of chronic cough in children. Prolonged acute cough is usually related to the long-lasting effects of a previous viral airway infection or to the particular entity called protracted bacterial bronchitis. Cough pointers and type may orient toward specific etiologies, such as barking cough in croup or tracheomalacia, paroxystic whooping cough in Pertussis. Cough is productive in protracted bacterial bronchitis, sinusitis or bronchiectasis. Cough is usually associated with wheeze or dyspnea on exertion in asthma; however, it may be the sole symptom in cough variant asthma. Thus, pediatric cough has particularities differentiating it from adult cough, so the approach and management should be developmentally specific.

## Introduction

### The cough reflex

Cough and expiration reflex are vital airway defensive mechanisms. Cough is traditionally defined as a 3-phase motor act characterized by an inspiratory effort (inspiratory phase) followed by a forced expiratory effort against a closed glottis (compressive phase) followed by opening of the glottis and rapid expiratory airflow (expulsive phase). The expiration reflex differs from the cough reflex in that it lacks the preparatory inspiration (Widdicombe and Fontana, 2006). The different motor pattern indicates these reflexes may have different neural control mechanisms and appear to subserve different functions (Widdicombe and Fontana, 2006). Reflex cough is mediated by both myelinated Aδ and unmyelinated C fibers originating from the nodose and jugular vagal ganglia (Canning et al., 2004), respectively, with a first relay station at the level of the medulla oblongata (Kubin et al., 2006). Little is known regarding the supramedullary structures involved in cough mediation. The neurophysiology of the expiration reflex has been even less extensively studied.

### Cough as a symptom and a disease

From a clinical point of view, pediatric cough is a symptom that shares many similarities but also differs in many ways from adults (Chang, 2010). Cough can be acute or chronic. In adults, chronic cough, i.e., lasting longer than 8 weeks (Morice et al., 2004), is a major and disabling symptom of many respiratory and non-respiratory diseases. Asthma syndromes, upper airway disorders and gastro-esophageal reflux disease (GORD) account for the majority of chronic cough cases (Morice et al., 2004), though in some instances no definite causative factor can be identified, i.e., idiopathic cough (Morice et al., 2004). Rather than a mere accompanying symptom, chronic cough has recently been proposed as a distinct clinical entity—“chronic cough hypersensitivity syndrome”—to account for its induction by triggers that would otherwise elicit no response in the normal population (Morice, 2010). The concept is captivating and may offer, at least in the long term, some advantages for patient management. In particular, establishing a single diagnostic entity might promote greater effort to develop novel and specific therapeutic strategies.

In children, several types of cough may be defined. *Acute* cough, typically lasting less than 2 weeks, is considered a mere symptom most frequently related to a respiratory tract infection. Cough lasting 4–8 weeks is increasingly recognized as “prolonged acute cough” mostly in relation to *protracted bacterial bronchitis* (Craven and Everard, 2013). *Chronic* cough lasts more than 4 weeks in children (Chang et al., 2014; Weinberger and Fischer, 2014) vs. 8 weeks in adults (Chang, 2010), and the quality—dry or productive, the latter suggesting more serious condition—must be specified. This cough may be associated to other signs or symptoms, called “cough pointers,” thus cough may be specific or non-specific, depending on the presence or absence of pointers.

## Prevalence, morbidity, and quality of life

In contrast to adults, assessing cough in children is rather difficult due to the lack of objective methods to measure cough frequency and intensity; subjective scoring methods are biased by parental perception of the child's symptoms (Dales et al., 1997).

Twenty two percent of preschool children report chronic cough without cold (Luyt et al., 1993) and the prevalence is up to 50% in children with 2 smoking parents (Charlton, 1984). It is higher in damp homes, polluted areas and low socioeconomic status (de Jongste and Shields, 2003).

Chronic cough is associated with high morbidity in children and their families. In contrast to adults, the child with chronic cough does not report anxiety or depression, but parents are often stressed, frustrated and unable to cope with their children's symptoms and their own sleepless nights (Marchant et al., 2008). When forceful, cough may determine symptoms like: syncope (Haslam and Freigang, 1985), pneumo-mediastinum, pneumothorax, urinary incontinence, or vomiting. It decreases the quality of life and affects daily activities, being responsible of significant absenteeism at school (Marchant et al., 2008).

Urge to cough is a respiratory sensation, allowing individuals to perceive the cognitive urge of cough reflex. This perception has important behavioral implications in cough motor action. Studies have suggested different methods to measure urge to cough in adults (Davenport et al., 2007; Kanezaki et al., 2012) and indicate its role in the volitional control of reflex cough (Davenport et al., 2009). Almost all studies have employed scoring methods originally developed for the assessment of dyspnea. In children, specific tools—especially pictorial scales—are emerging for the monitoring of dyspnea (Yelling et al., 2002), but there are currently no data on pediatric urge to cough. Cough and dyspnea may exhibit some similarities as they are both nociceptive signals (Gracely et al., 2007). Respiratory afferents and brain structures responsible for integration into a perception of urge to cough may therefore resemble those involved in dyspnea. Afferent pathways of dyspnea are complex, but sensations like chest tightness are mediated by afferent fibers traveling through the vagus nerve, similar to cough receptors (Gracely et al., 2007). A likely hypothesis is that urge to cough—like dyspnea—results from the cortical processing of these afferent messages, and the unconfortable perception may or not trigger the motor act of cough. It may be hypothesized that developmental specificities, demonstrated for instance by the blunted discharge of vagal afferents in the neonatal animal (Sant'Ambrogio, 1982) or the pediatric characteristics of dyspnea (Von Leupoldt et al., 2006) may similarly impact on the perception of the urge to cough in children. The development of specific tools to assess urge to cough in daily care is justified by the incidence of chronic cough in pediatrics. The lack of dyspneic sensation has been shown to contribute the genesis of life-threatening asthma in children (Kifle et al., 1997). This points out the importance of nociceptive perception in children. The long-term impact of pain on behavior and/or psychophysics of pain perception during development have received some attention (McEwen, 1998; Buskila et al., 2003), but no such data exist for dyspnea or other nociceptive respiratory sensations such as the urge to cough in children.

Chronic cough in children is strongly related to affective state including stress and frustration resulting in sleepless nights for families, but there are unfortunately little data on the long term impact of respiratory sensations on the child's development and behavior, partly because of lack of appropriate testing methods.

## Specific cough

Cough quality may orientate toward specific etiologies (Table 1) (Chang and Glomb, 2006) and pointers that help diagnosing specific conditions are shown in Table 2.

**Table 1:** **Cough quality and etiology**.

**Cough quality**	**Etiology**
Barking or brassy	Croup
	Tracheomalacia
Honking	Habit, psychogenic cough
Paroxystic	Pertussis
Associated or not to whoop	Parapertussis
Dry, staccato	Chlamydia infection in infants
Productive	Sinusitis
	Protracted bacterial bronchitis
	Bronchiectasis

**Table 2:** **Cough pointers and etiology**.

**Cough pointers**	**Etiology**
Wheeze Dyspnea on exertion Atopy	Asthma
Hemoptysis	Interstitial lung disease
	Bronchiectasis
Chest deformity	Chronic lung disease
Penetration syndrome	Foreign body inhalation
Symptom worsening after feeding or when lying down	GORD
	Pulmonary aspiration
Auscultatory findings	Chronic lung disease
Digital clubbing	Systemic immunodeficiency
Failure to thrive	Cardiac disease
Medical history of prematurity and neonatal respiratory intensive care	Bronchopulmonary dysplasia
Symptoms present from the first day of life	Congenital abnormalities

## Non-specific cough

In adults, upper airway diseases, asthma with related syndromes and GORD predominate (Morice et al., 2004) while in children, postinfectious cough, protracted bacterial bronchitis (Chang et al., 2014; Weinberger and Fischer, 2014) and asthma are the main causes. Furthermore, in children, the etiology is age-dependent: bronchiolitis and croup up to 2 years of age, postinfectious and foreign body inhalation at preschool (Chang et al., 2006a) or school age, habit cough in adolescent (Berman, 1966).

### Upper airways diseases

Have a high prevalence in children, up to 14.6% (Berman, 1966) and have been consistently reported as a major cause for chronic cough (20–40%) also among adult patients (Morice et al., 2004). The pathogenesis of these cough syndromes is complex, involving a combination of microaspiration, post nasal drip of mucus, spreading of inflammation via systemic circulation, naso-bronchial reflex, sino-bronchial reflex, lack of nasal functions in subjects with rhinitis together with mechanisms of central and peripheral cough plasticity (Plevkova and Song, 2013). During a common cold, patients are experiencing bouts of coughing as the result of minor environmental insults such as change in air temperature or exposure to noxious stimuli like cigarette smoke. Objective evidence of this “virus induced” cough hypersensitivity relies on challenge experiments (O'Connell et al., 1996; Plevkova et al., 2006; Dicpinigaitis et al., 2011), explaining the virus ability to disseminate through the population using droplet transmission (Morice, 2013). It was assumed that cough is induced by airway afferent nerves expressing TRPV1 and TRPA1 channels, respiratory viruses up-regulating cough reflex via these TRP channels. The increase in TRPA1 and TRPV1 levels (implicated in up-regulation) can be mediated by soluble factors induced by infection whereas TRPM8 (menthol receptor for down-regulation) requires replicating virus. It was hypothesized that viruses regulate cough by their replication cycle inside the host organism (Plevkova et al., 2013). These informations are appealing as nowadays, TRP channels appear to be very promising targets to manage cough (Abdullah et al., 2014).

### Cough and GORD

In adults the role of GORD in chronic unexplained cough is widely accepted (Irwin, 2006; Pacheco-Galvan et al., 2011) and the benefit of GORD treatment is indicated (Irwin et al., 1998; Irwin and Madison, 2002; Morice et al., 2004). Several studies in children revealed that unexplained chronic cough ascribed to acid reflux ranges 15–45% (Holinger and Sanders, 1991; Jain et al., 2002; Thilmany et al., 2007; Khoshoo et al., 2009). Other studies advocate only the co-incidence of cough with symptoms of GOR (Nelson et al., 1998; Chang et al., 2006b). The evaluation of the cough—acid reflux relationship using esophageal pH monitoring and a cough logger did not detect any temporal association between the two conditions (Chang et al., 2011). The relation of chronic unexplained cough to GORD thus remains controversial in children and pediatric guidelines do not currently recommend empirical GORD treatment trials for pediatric chronic cough (Chang et al., 2006a). Recently, expanding the diagnostic power of pH-metry by combining with esophageal impedance monitoring suggested that cough episodes were frequently preceded by weak acid reflux (Pauwels et al., 2009; Borrelli et al., 2011; Ghezzi et al., 2013) especially in children aged less than 2 years (Ghezzi et al., 2013). The potential role of weak acid reflux in chronic cough could explain why cough reflex sensitivity to capsaicin was significantly enhanced not only in children with GORD diagnosed by 24 h pH monitoring but also in a group of children with symptomatic but not confirmed GORD (Varechova et al., 2007a).

GOR may trigger cough by two major mechanisms: (a) acute or chronic (micro-) aspiration of refluxed material into the airways or (b) by the activation of the vagal afferent nerves in the esophagus that may directly trigger cough or, more likely, may lead to cough reflex up-regulation at central (brainstem) level. Animal studies have identified and characterized the neural pathways for two putative mechanisms, reviewed in a recent paper (Kollarik and Brozmanova, 2009).

### Cough and asthma

Cough and bronchoconstriction reflexes are physiological mechanisms important to preserve the integrity of the respiratory system. Inflammatory processes of the respiratory tract are associated with up regulation of both—cough hyperactivity and bronchial hyperactivity—and may lead to asthma or be associated with chronic cough. The evidence of asthma is based on the joint occurrence of airway inflammation and airway smooth muscle hyperresponsiveness. Numerous pro-inflammatory mediators released in asthmatic airways activate primary afferent neurons. In this context of peripheral neuronal sensitization, cough may be a symptom of asthma exacerbation. Indeed, several studies have shown a close relationship between cough reflex sensitivity and eosinophilic infiltration of the airways when treatment with inhaled steroids decreases the cough sensitivity to inhaled citric acid in patients with asthma (Di Franco et al., 2001). Chang and co-workers observed that in asthmatic children with troublesome cough, the cough reflex sensitivity is enhanced during the acute severe phase but decreases thereafter to levels similar to those in children with asthma but no cough. This study is in keeping with the clinical observation in children that cough frequently heralds an asthma exacerbation (Chang et al., 1997a). Cough variant asthma is a particular phenotype characterized by cough rather than wheeze as sole symptom, and bronchial hyperresponsiveness is demonstrated by a positive methacholine challenge. This variant of asthma responds to bronchodilator, inhaled steroids or antileukotriene therapy (Niimi, 2013) and shares some typical features of allergic asthma such as the presence of atopy, milder eosinophilic airway inflammation and airway remodeling (Niimi et al., 2000; Niimi and Chung, 2004).

## Physiological mechanisms of cough during development

### Cough during development

The cough reflex appears to be modulated throughout postnatal development. There are no data on cough recording in the human fetus (Chang and Widdicombe, 2007) and at birth, cough seems to be absent, or rarely present as part of the laryngeal chemoreflex (Thach, 2007). As maturation progresses, cough response to stimulation by fluid contact with the laryngeal mucosa increases (Perkett and Vaughan, 1982; Pickens et al., 1988), cough being reported below 1 month of age in infants with respiratory tract infection (Schaad and Rossi, 1982) or foreign body inhalation (Singh et al., 1999). Cough becomes the most frequent respiratory symptom during the first years of life, but objective data on incidence of the cough reflex in that age range are lacking, mainly because infants and young children lack the active cooperation required to perform capsaicin sensitivity test. That cough- and expiration reflex may undergo a maturational process during childhood is also suggested by experimental studies.

Several types of animal models, behaving or anesthetized, are used to clarify cough mechanisms and develop new therapies. In the behaving animal, cough may be chemically induced by capsaicin or citric acid similar as in human. The advantage of using an anesthetized animal is to apply mechanical stimuli at different levels of the airways and during different phases of the respiratory cycle in order to differentiate cough and expiratory reflex. At birth, many animal species, except cat (Korpas and Tomori, 1979), do not cough in response to laryngeal or tracheobronchial stimulation, while the expiration reflex, apnea or other defensive reflexes are observed (Harding et al., 1978; Sutton et al., 1978; Korpas and Tomori, 1979; Thach, 2001). Cough reflex develops afterwards. Studies of the response to laryngeal stimulation in waking animals indicate that cough regularly observed in adult dogs (Sullivan et al., 1978) is mostly missing in the newborn lamb where apnea is the major response (Marchal et al., 1982).

Most airway afferent nerve subtypes modulate cough reflex. Slowly adapting receptors that facilitate the cough reflex in adults (Karlsson et al., 1988) have a low rate of discharge in newborn mammals (Sant'Ambrogio, 1982), which may contribute to limit the occurrence of cough. In the neonatal dog, rapidly adapting receptors are scarce (Fisher and Sant'Ambrogio, 1982), also suggesting decreased ability to cough. Therefore, these developmental changes in lung afferent characteristics may impact the cough reflex in infancy. Hypersensitivity of C-fiber to capsaicin demonstrated in weanling rats following an experimental respiratory syncytial virus infection suggests a role for the virus in triggering apnea in infants with bronchiolitis (Peng et al., 2007). Similar up regulation could explain the prolonged cough that sometimes follows bronchiolitis. It is difficult to assess how these characteristics of afferent discharge impact urge to cough sensation or cough reflex. These processes result from central integration of vagal afferent traffic and there are no data during childhood.

Later during growth, the objectively measured incidence of cough seems to decrease from school-age, 1–34 coughs/24 h (Munyard and Bush, 1996), to adulthood, 0–16 coughs/24 h (Hsu et al., 1994). This is in agreement with studies assessing cough sensitivity to capsaicin that decreases from prepubertal stage to mid-puberty and it decreases further in boys, but increases in girls at late puberty (Varechova et al., 2008). Indeed, in adulthood, higher cough frequency and increased cough sensitivity have been repeatedly reported in females compared with males, both healthy volunteers (Fujimura et al., 1990, 1996; Dicpinigaitis and Rauf, 1998) and patients with chronic cough (Os et al., 1992; Morice et al., 2000; Kastelik et al., 2002). The unique anatomy of their oeso-gastric junction makes humans prone to reflux and aspiration (Pacheco-Galvan et al., 2011) and female cough hypersensitivity has been suggested as evolutionary mechanism that protects against aspiration during pregnancy (Morice, 2013).

### Plasticity of the cough reflex

There is considerable evidence that neural pathways regulating cough may undergo short- or long-term changes in structure and function—referred to as plasticity—in response to physiological conditions such as breathing, swallowing and other respiratory reflexes, sleep or exercise (Marchal et al., 1987; Lavorini et al., 2010; Varechova et al., 2010), environmental exposures and pathological processes (Chang et al., 1997b). In the case of acute cough due to upper airway infection, non-specific chronic cough associated with GORD, upper airway diseases or cough variant asthma (see above) or cough due to environmental tobacco smoke (Joad et al., 2004, 2007), the protective cough reflex may be replaced by exaggerated coughing to subthreshold stimuli (Bonham et al., 2006). Mechanisms of cough sensitization may be of peripheral or central origin, where continued presence of numerous irritants, inflammation and tissue damage can lead to increased excitability of nerve terminal thus lowering its threshold for activation (McAlexander and Carr, 2009) as well as to de novo substance P expression in mechanosensitive Aδ vagal fibers (Myers et al., 2002). The release of substance P on peripheral terminal in airways and central terminal in nucleus tractus solitarius (NTS) can contribute to increased sensory traffic to NTS and even to intrinsic cell hyperexcitability of NTS neurons and heightened cough response.

The aspect of developmental plasticity is fascinating as can relate greatly to pediatric cough but needs further investigation. Several studies have shown that exposing young guinea pigs to second hand tobacco smoke increased citric acid cough and exposition to ozone alone or in combination with allergen increased intrinsic excitability of second-order neurons in the NTS in infant non-human primates. This points to the fact that cough reflex pathway may undergo plasticity process as a result of a strong interplay between the developing organism and pre-natal and/or post-natal environmental conditions.

There are many conditions in which cough can be down-regulated in health and disease, by the mechanisms that interact at the level of cortical control for cough and/or by the secondary afferent inputs from bronchopulmonary, chest wall, limb or visceral receptors. In disease, cough reflex sensitivity to capsaicin is decreased in children with cystic fibrosis compared to healthy children, where altered quality of airway mucus, bacterial colonization of airways may cause tachyphylaxis of cough receptors (Chang et al., 1997b). A similar decrease was observed in diabetic children with subclinical diabetic autonomic neuropathy (Varechova et al., 2007b) that can account, at least partially, for increased incidence of respiratory infections in these children. Therefore, elucidating the various mechanisms of down-regulation could be worth in the context of developing novel antitussive therapy.

Defensive airway reflexes such as cough, sneeze or apnea are motor acts replacing the normal breathing pattern in order to protect/defend the airways from potentially harmful agents. Repetitive strong inspirations (during cough attack, aspiration reflex) result in hyperventilation, whereas apnea and a series of expiration reflexes may cause hypoventilation (Korpas and Tomori, 1979). Airway defensive responses are modulated during breathing (Varechova et al., 2010). For instance, cough reflex is the frequent response to a punctuate tracheal stimulus delivered during inspiration, while expiration reflex is the typical response in expiration. Cough is downregulated when induced during a reflex apnea (Poussel et al., 2012), but appears unaffected during apnea induced by hyperventilation (Tomori et al., 1991). Simultaneous tracheobronchial and laryngeal stimulation results in increased coughs (Kondo and Hayama, 2009). Interaction of motor acts in particular reflex responses is characterized by their replacement (substitution), yielding (allowing), potentiation and, sometimes, overlapping.

## Conclusive statements—what do we need to know?

Chronic cough is frequent in childhood and should be treated based on etiology. The true prevalence of cough is difficult to evaluate given the methodological difficulties (Chang et al., 1997a). There is no objective method to measure cough in children and instruments designed for the adult require modification for use in children (Corrigan and Paton, 2003). Cough-specific QOL questionnaires exist for adults but not children and adult QOL scores cannot be applied to children (Chang, 2005). In the near future, there will be a need for more precise and reliable methods to measure the cough reflex sensitivity and improve our understanding of the mechanisms underlying up-regulation of cough, increase the knowledge on effect of maturation on cough reflex to develop new treatment strategies adapted to the child's age.

### Conflict of interest statement

The authors declare that the research was conducted in the absence of any commercial or financial relationships that could be construed as a potential conflict of interest.
